# Dendritic Cells and Myeloid Derived Suppressor Cells Fully Responsive to Stimulation via Toll-Like Receptor 4 Are Rapidly Induced from Bone-Marrow Cells by Granulocyte-Macrophage Colony-Stimulating Factor

**DOI:** 10.3390/vaccines8030522

**Published:** 2020-09-12

**Authors:** Ying Ying Kong, Kirsty Wilson, Vasso Apostolopoulos, Magdalena Plebanski

**Affiliations:** 1 Institute for Molecular and Cellular Biology, Agency for Science, Technology and Research (A*STAR), Singapore 138673, Singapore; kongyingying@tessatherapeutics.com; 2 School of Health and Biomedical Sciences, RMIT, Bundoora, VIC 3083, Australia; kirsty.wilson2@rmit.edu.au; 3 Institute for Health and Sport, Victoria University, Melbourne, VIC 3011, Australia

**Keywords:** granulocyte-macrophage colony-stimulating factor, GM-CSF, dendritic cells, myeloid derived suppressor cells, MDSCs, bone marrow culture

## Abstract

Dendritic cells (DCs) are commonly generated from bone marrow (BM) progenitor cells with granulocyte-macrophage colony-stimulating factor (GM-CSF) alone or in combination with interleukin 4 (IL-4). These cells are often harvested post day 5, when they acquire maturation markers and can stimulate T cells. Apart from DCs, myeloid derived suppressor cells (MDSCs) are also found within these cultures. However, little is known about the functional characteristics of DCs and MDSCs before day 5. Herein, using a murine model, it is shown that early DCs and MDSCs, even in cultures with GM-CSF alone, upregulate fully maturation and activation surface molecules in response to the toll-like receptor 4 (TLR4) ligand lipopolysaccharide (LPS) stimulation. Despite initially displaying lower marker expression levels, these cells efficiently induced T cell stimulation and cytokine production. Interestingly, Gr-1^int^ MDSCs increased their T cell co-stimulatory activity upon TLR4 stimulation. Additionally, early DCs and MDSCs exhibited differential endocytic capacity for viral sized nanoparticles and bacterial sized microparticles. DCs internalized both particle sizes, whilst MDSCs only internalized the larger microparticles, with reduced endocytic activity over time in the culture. These findings have unveiled an important role for the rapid initiation of productive immunity by GM-CSF, with promising implications for future vaccine and DC immunotherapy developments.

## 1. Introduction

The bone marrow (BM) is the primary source of key immunostimulatory antigen presenting cells (APC) such as dendritic cells (DCs), as well as innate regulatory cells such as myeloid derived suppressor cells (MDSCs). DCs link the innate and adaptive immune system by recognizing pathogens and presenting peptide epitopes to T cells. They are the most efficient APC, able to prime naïve T cells. Although scarce in number, DCs are strategically located in most tissue niches, which allows them to interact proficiently with T cells and B cells [1]. DCs all have in common the ability to recognize pathogens, capture and process antigens, and present them to T cells to activate the adaptive immune response [1,2,3]. This process requires three signals: recognition of the peptide epitope in the major histocompatibility complex (MHC) by the T cell; expression of co-stimulatory molecules; and secretion of cytokines. Immature DCs typically express low to moderate levels of the co-stimulatory molecules CD80 (B7-1), CD86 (B7-2), CD40 and MHC class I and II [2]. When fully activated they are capable of secreting T helper (Th)1 polarizing cytokines such as interleukin (IL)-12, tumor necrosis factor (TNF) and interferon (IFN)-γ [4,5], and Th2 polarizing cytokines such as IL-4, IL-5, IL-6 and IL-13 [6].

Conversely, MDSCs are a heterogeneous population of early myeloid progenitors which can exert immuno-regulatory functions, and consist of immature granulocytes, macrophages and DCs [1,2,3]. These cells consistently express the surface integrin CD11b as well as varying levels of the myeloid lineage differentiation antigen, Gr-1 (Ly6G/Ly6C) [3,4,5]. MDSCs can be divided into two subsets: monocytic and granulocytic MDSCs. Several murine studies have identified additional markers such as F4/80, intracellular adhesion molecule 1 (ICAM-1) and C-C chemokine receptor 2 (CCR2) that are also expressed on MDSCs [3,6,7]. MDSCs, most prominently monocytic MDSCs, inhibit T cell activation and prevent antigen presentation of APCs by producing nitric oxide, reactive oxygen species (ROS) and arginase-1 [8,9].

The hematopoietic growth factor granulocyte-macrophage colony-stimulating factor (GM-CSF) has been commonly used to generate both DCs and MDSCs from BM cells in vitro. Administered intravenously in humans it is also capable of attracting monocytes, DC and MDSC precursors into circulation, where they can be captured to further expand them for diverse applications, for example, re-infusible DC-based vaccine therapies [10]. GM-CSF is a pro-inflammatory cytokine thought to be secreted by lymphocytes involved in the development of myeloid cells. GM-CSF is implicated in inflammatory conditions, due to its upregulation and ability to attract macrophages and direct monocytes into inflammatory type DCs during infection [11,12]. There have also been clinical benefits for some autoimmune and inflammatory conditions by blocking GM-CSF and/or the GM-CSF receptor [13].

GM-CSF BM cultures are heterogenous and include both functional DCs and macrophages with distinct marker expression and capabilities [14,15]. Other myeloid and granulocytic cell types may also be present in these cultures. BM-derived DCs grown in GM-CSF are highly phagocytic and have a survival advantage compared to steady state cells grown using fms-like tyrosine kinase 3 (FLT-3) ligand [16]. Lipopolysaccharide (LPS) has also been shown to increase the survival of GM-CSF derived DCs, mediated by the phosphoinositide 3-kinase (PI3K) pathway [17].

The functionality of BM cell derived DCs or MDSCs in early GM-CSF stage cultures has not been fully defined. Previously it has been shown that early stage (Day 3 or 4) GM-CSF derived DCs are functional, though their cytokine secretion and T cell stimulatory capacity are reduced in the presence of cancer cells [18]. Recent studies also suggest that the induction of mature inflammatory DCs by GM-CSF may also inadvertently induce MDSCs, thus potentially impeding the efficacy of DC-based vaccines and immunotherapies. This led us to investigate the properties and functions of DCs and MDSCs generated in early GM-CSF derived BM cultures (Day 3–5). Herein we use a murine model to characterize these cells and show that early DCs and MDSCs are capable of rapidly responding to pro-inflammatory stimuli to activate and undergo maturation, expressing high levels of T cell co-stimulatory molecules, whilst retaining endocytic properties. Importantly, early DCs post LPS maturation efficiently induce T cell proliferation, whereas early culture monocytic MDSCs do not induce T cell proliferation unless in the presence of the T cell stimulation molecules anti-CD3 and anti-CD28. Together these data shed new insight into the biology of DCs and MDSCs and suggest that these cell types are differentially regulated via the toll-like receptor 4 (TLR4) receptor to promote an overall pro-inflammatory environment.

## 2. Materials and Methods

### 2.1. Animals

C57BL/6 mice 6–8 weeks old were obtained from the Precinct Animal Centre (PAC) at the Alfred Medical Research and Educational Precinct (AMREP) (Melbourne, VIC, Australia). BALB/c mice aged between 6–8 weeks were obtained from Monash Animal Services (Melbourne, VIC, Australia). All experiments had ethics approval by the Animal Ethics Committee of AMREP under ethics numbers E/1032/2010/M, E/1052/2011/M and E/1130/2011/M.

### 2.2. Isolation of BM Cells from Mice

Mice were culled by CO_2_ asphyxiation. The mice were sprayed with 70% *v/v* ethanol to prevent bacterial contamination, and skin removed to expose the legs. Femur and tibia of both legs were extracted, and muscles removed. The bones were then soaked in 70% *v/v* ethanol for 1 min to ensure aseptic conditions. Bones were removed from ethanol and thoroughly washed before being moved to a fresh tube of sterile RPMI (supplemented with 10% fetal bovine serum (FBS), 2 mM L-glutamine, 20 mM HEPES, 0.1 mM 2 mercaptoethanol and 100 units/mL penicillin and 100 μg of streptomycin; complete media; CM). Both ends of each bone were carefully cut off to expose the BM. A 3 mL syringe with 25-gauge needle filled with CM was used to flush each bone to dislodge the BM. The cells were then disaggregated with a 1 mL pipette and filtered through a cell strainer (100 μm, Millipore, Billerica, MA, USA) into a 10 mL centrifuge tube. The cells were centrifuged at 1400 rpm for 5 min at room temperature. The supernatant was carefully removed, and BM cells (with erythrocytes) were re-suspended in 1 mL of Ammonium-Chloride-Potassium (ACK) lysis buffer for 1 min to lyse erythrocytes. Lysis buffer was diluted with 9 mL of CM and centrifuged again at 1400 rpm for 5 min at room temperature. The supernatant was removed carefully and the BM cells (without erythrocytes) were re-suspended in 10 mL of CM.

### 2.3. GM-CSF Derived DC Culture

The concentration of BM cells was adjusted to 5 × 10^5^ cells/mL in CM. GM-CSF (PeproTech, Rocky Hill, NJ, USA) was added to the cell suspension at a final concentration of 10 ng/mL. Where stated, IL-4 (PeproTech, Rocky Hill, NJ, USA) was also added to the cultures at a final concentration of 5 ng/mL. BM cells were cultured in 24-well plates in 1 mL of CM with GM-CSF or GM-CSF + IL-4 and incubated at 37 °C in 5% CO_2_. Cells were harvested by gentle resuspension on either day 3, 4 or 5 unless otherwise stated. To harvest the cells, the plates were centrifuged at 1400 rpm for 5 min at 4 °C. The supernatant was collected, and cells were re-suspended in phosphate buffered saline (PBS) and prepared for cell surface staining.

### 2.4. DC Activation by Lipopolysaccharide

Where indicated, cells on culture days 3, 4 or 5 were co-cultured with or without LPS (1 μg/mL, derived from Escherichia coli; 0111:B4, Sigma-Aldrich, Louis, MO, USA) and incubated a further 24 h at 37 °C in 5% humid CO_2_ atmosphere. After 24 h, the plates were centrifuged, the supernatants were collected, and the cells harvested by gentle resuspension, for analysis by flow cytometry.

### 2.5. Preparation and Incubation of Fluorescent Particles in BM Culture

AF488-labelled carboxylate-modified polystyrene microspheres (0.04 μm (F8795, 5% solids in water, Lot # 41892A, and 0.5 μm carboxylate-modified polystyrene microspheres F8813, 2% solids in water + azide, Lot # 23115W, Invitrogen-Molecular Probes, Carlsbad CA, USA) were dialyzed in MilliQ water overnight and sonicated for 15 min to reduce aggregation before using. 40 nm fluorescent particles (8 × 10^4^ particles/cell) and 500 nm fluorescent particles (51.2 particles/cell) were diluted in CM and added into the culture for 1 h before the cells were harvested by gently resuspending the culture. The particle uptake by cultured cells was analyzed by flow cytometry, measuring the intensity of the AF488 stain.

### 2.6. Fluorochrome-Conjugated Antibody Cocktail Preparation and Cell Surface Staining

All Fluorochrome-conjugated antibodies were titrated beforehand to determine optimal dilutions for detection by flow cytometry. The cells were harvested and stained with a combination of fluorochrome-conjugated antibodies (Table 1). Dead cells were discriminated using the LIVE/DEAD Fixable Aqua Dead Cell Stain Kit (Invitrogen, Life Technologies, Carlsbad, CA, USA). Antibodies were prepared in flow cytometry staining buffer (PBS + 2% *v/v* FBS) and cells were stained for 20 min on ice in the dark. After incubation, the cells were washed with staining buffer and centrifuged at 1400 rpm, at 4 °C for 5 min. The supernatant was carefully removed, and the cells were re-suspended in 100 μL of PBS/1% *v/v* paraformaldehyde. Samples were acquired with the LSRII (BD Biosciences, Franklin Lakes, NJ, USA) at the AMREP Flow Cytometry Core Facility (AMREP, Melbourne, Victoria, Australia). The data were analyzed using FlowJo Flow Cytometry Analysis Software (TreeStar Inc., Ashland, OR, USA). Cell clumps, dead cells and debris were removed during analysis on basis of the forward scatter/side scatter (FSC/SSC) and the Fixable Aqua Dead Cell Stain Kit to isolate live DCs; the percentage of live cells and the mean fluorescence intensity (MFI) and/or the percentage of marker expression were measured.

### 2.7. T Cell Purification

Splenocytes from naïve BALB/c mice were harvested and incubated with ACK lysis buffer for 3 min at RT to remove erythrocytes. T cells from splenocytes were isolated based on the manufacturer’s instructions using the MACS^®^ Pan-T cells isolation kit (Miltenyi Biotec, Bergisch Gladbach, Germany). In brief, remaining cells were washed and incubated with a cocktail of biotin-conjugated antibodies against CD11b, CD11c, CD19, CD45R (B220), CD49b (DX5), CD105, MHCII, and Ter-119 for 10 min on ice and further incubated with anti-biotin magnetic beads for 15 min at 4 °C. The isolation of unlabeled T cells was achieved by depletion of magnetically labelled cells. 

### 2.8. Mixed Leukocyte Reaction Assay

To evaluate the T cell stimulatory capacity of DCs derived from GM-CSF BM cultures at day 3 or 4, cultured cells were either left un-stimulated or stimulated with LPS and incubated for another 24 h at 37 °C in 5% humid CO_2_ atmosphere before they were harvested and stained with either anti-CD11c V450 (HL3), Gr-1 PerCP Cy5.5 or biotin Gr-1 (RB6-8C5)-Streptavidin-AlexaFluor 700 for 20 min on ice in the dark. The cells were then washed and re-suspended in PBS with 2% *v/v* FBS, 0.01 M of EDTA at a concentration of 10^7^ cells/mL and sorted with a cell sorter (BD Influx, BD Biosciences, Franklin Lakes, NJ, USA) at the AMREP Flow Cytometry Core Facility. DCs, granulocytic MDSCs and monocytic MDSCs were sorted based on CD11c+ Gr-1-, CD11c- Gr-1^hi^ and CD11c- Gr-1^int^ expressions, respectively. Sorted BALB/c T cells (10^5^ cells) were stained with CellTrace™ carboxyfluorescein succinimidyl ester (CFSE) cell proliferation kit, as per manufacturer’s instructions, (Molecular Probes, Eugene, OR, USA) and seeded into 96 well U-bottom plates with the sorted cells (3 × 10^3^ cells/well) in triplicates and incubated for three days at 37 °C in 5% humid CO_2_ atmosphere. T cell proliferation was analyzed by measuring the MFI of CFSE via flow cytometry using the LSRII.

### 2.9. T Cell Co-Stimulation Assay

To assess the ability of cells isolated from day 3 or 4 GM-CSF derived BM cultures to induce T cell proliferation in the presence of an added stimulus, the cells were either left un-stimulated or stimulated with LPS for 24 h before they were harvested and sorted as described above. Sorted BALB/c T cells (10^5^ cells) were stained with CellTrace™ CFSE cell proliferation kit and seeded with sorted cultured BM cells (3 × 10^3^ cells). Soluble anti-CD3 (5 μg/mL) and anti-CD28 (5 μg/mL) (anti-CD3/28, eBioscience, Inc., San Diego, CA, USA) were added and the culture was incubated for three days at 37 °C in 5% humid CO_2_ atmosphere. T cell proliferation was analyzed by flow cytometry using the LSRII.

### 2.10. Cytokine Detection

To detect and quantify cytokines secreted by the cultured BM cells, supernatants from GM-CSF derived BM cultures, either un-stimulated or stimulated with LPS (1 µg/mL), incubated for 24 h at 37 °C in 5% humid CO_2_ atmosphere, were collected. Supernatants were analyzed using a cytometric bead array (CBA) inflammation kit (BD Biosciences, Franklin Lakes, NJ, USA), which included the cytokines IL-6, IL-10, monocyte chemoattractant protein 1 (MCP-1), and TNF. Preparation of the supernatants was performed following the manufacturer’s instructions. Briefly, for the CBA inflammation kit, 50 μL of either culture supernatant or standard cytokine solutions (0–5000 pg/mL) were incubated with 50 μL of cytokine capture beads and PE detection reagent for 2 h. Samples were acquired with the LSRII according to the CBA setup instructions in the kit. A minimum of 400 events/cytokine was acquired for each sample. Standard cytokine solutions included in the inflammation kit and flexsets were used to plot out the standard curves and quantitative analysis of cytokines was done using the FCAP Array software (Soft Flow Inc., St. Louis Park, MN, USA) based on the level of MFI.

### 2.11. Statistical Analyses

The data generated are shown as mean ± standard deviation (SD). All values were graphed and analyzed for statistical significance using Prism (GraphPad Software, Inc., San Diego, CA, USA). Student’s T-test was performed on two normally distributed experimental groups. For three or more experimental groups at a single time-point, one-way ANOVA with Tukey–Kramer multiple comparison tests was used if the data were normalized; if the data were not normalized, Kruskal–Wallis test with Dunnett’s multiple comparison test was used. Two-way ANOVA with Bonferroni post-tests was used for three or more experimental groups at two or more time-points. Statistical significance was indicated by the *p*-value (* denotes *p* < 0.05; ** denote *p* < 0.01, *** denote *p* < 0.001).

## 3. Results

### 3.1. GM-CSF Induces an Early Surge in DC Generation without Inducing Expression of Activation Markers in BM Cultures

In vitro DCs are generated from BM with various cytokine combinations such as GM-CSF, GM-CSF + IL-4 or FLT-3L among others over a period of 5–9 days [19,20,21]. Despite the wide range of possible cytokine combinations available for DC generation, GM-CSF remains the most used cytokine. Although these cultures have been widely studied for their immunogenicity, little is known about the DCs or other myeloid cells present at the earlier time-points (days 3–5) in these cultures. 

**Figure 1:**
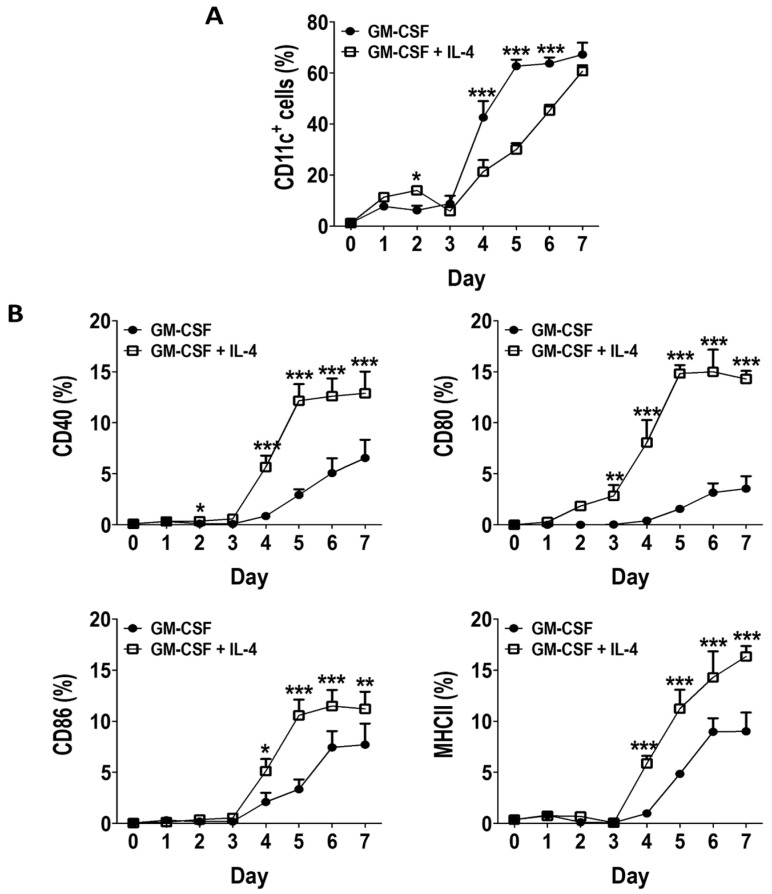
GM-CSF induces generation of DCs with lower activation marker expression in BM cultures compared to GM-CSF + IL-4. DCs (CD11c+ cells) generated from murine BM with GM-CSF (10 ng/mL) (black circles) or GM-CSF (10 ng/mL) + IL-4 (5 ng/mL) (white squares) from days 1–7 were evaluated for (**A**) the percentage of DCs generated and (**B**) the expression of CD40, CD80, CD86 and MHCII on the DCs. Results shown are from three mice, two-way ANOVA was used to compare both cultures at each time-point. Results displayed as mean +/− SD. * *p* < 0.05, ** *p* < 0.01, *** *p* < 0.001.

The generation of in vitro DCs (characterized by surface expression of CD11c) from BM was systematically investigated by culturing murine BM cells for up to 7 days with GM-CSF alone or with GM-CSF + IL-4, monitoring the proportion of DCs daily using flow cytometry, measuring surface expression of CD11c. DC generation in GM-CSF cultures peaked and plateaued on day 5, while DC generation in GM-CSF + IL-4 cultures was slower and steadily continued to increase from days 3 to 7 (Figure 1A). By day 7 both cultures contained about 60% DCs (CD11c+ cells, Figure 1A). Expression of molecules associated with a mature DC phenotype (CD40, CD80, CD86 and MHCII) were significantly lower on DCs generated in GM-CSF cultures when compared to GM-CSF + IL-4 cultures, even though we observed a gradual increase in expression of these molecules from days 4 to 7 (Figure 1B). These results suggest that GM-CSF derived BM cultures can rapidly generate DC-like cells in culture with a more immature phenotype then DCs generated from GM-CSF + IL-4 cultures.

### 3.2. Phenotype of GM-CSF Cultured Cells Yield Conventional DCs, Granulocytic Gr-1^hi^ and Monocytic Gr-1^int^ MDSCs

**Figure 2:**
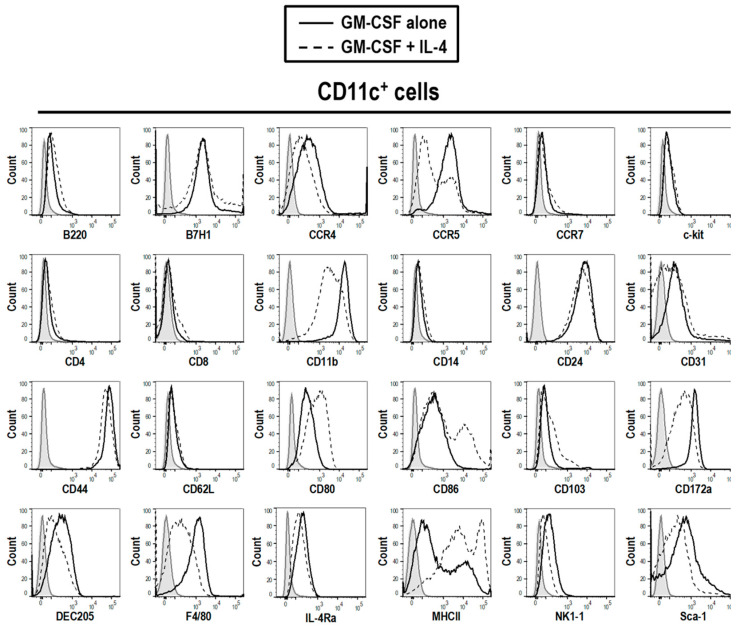
The phenotype of cells from GM-CSF treated BM cultures is consistent with the presence of DCs. Live cells from GM-CSF derived BM culture were characterized on day 3. Cells generated from BM with GM-CSF (10 ng/mL) (solid line) or GM-CSF (10 ng/mL) + IL-4 (5 ng/mL) (dotted line) were characterized with a panel of phenotypic markers, chemokine receptors and activation markers. The shaded grey area represents unstained control cells. Results shown are representative of three mice.

The cells isolated from the BM cultures generated using either GM-CSF alone or GM-CSF + IL-4 were tested for differential expression of surface makers by determining the MFI within the CD11c+ population (Table 2). These GM-CSF derived DCs expressed lower levels of activation markers CD80 (751.3 ± 10.79), CD86 (991 ± 26.96), MHCII (2052 ± 248.6) than their GM-CSF + IL-4 counterparts (CD80, 1368 ± 13.5; CD86, 3229 ± 427; MHCII 12,634 ± 452.3) (Table 2). As expected, DCs derived from either culture, GM-CSF or GM-SCF + IL-4, exhibit a conventional DC phenotype, expressing CD11b but not B220, CD4 or CD8 (Figure 2). The DCs also expressed high levels of CD24 and CD44, which are associated with regulating T cell functions [22,23]. Based on the observations above, it can be postulated that GM-CSF derived DCs are conventional myeloid DCs that are less mature but may still be able to regulate T cell functions.

**Figure 3:**
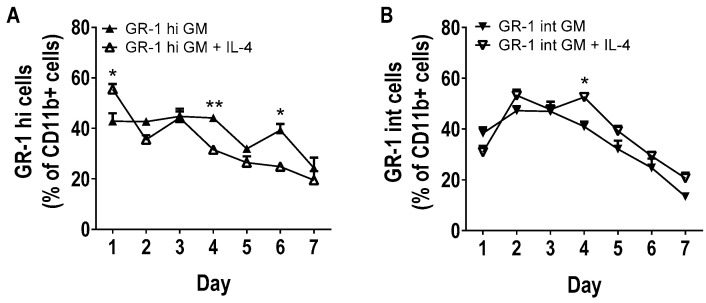
GM-CSF and GM-CSF + IL-4 cultures induce the generation of two subsets of MDSC. MDSCs (CD11c-, CD11b+, Gr-1+ cells) generated from murine BM with GM-CSF (10 ng/mL) (closed triangles) or GM-CSF (10 ng/mL) + IL-4 (5 ng/mL) (open triangles) from days 1-7. Cells were assessed for the percentage of (**A**) Gr-1^hi^ MDSCs and (**B**) Gr-1^int^ MDSCs generated within CD11c-, CD11b+ cells. Results shown are from three mice, two-way ANOVA was used to compare both cultures at each time-point. Results displayed as mean +/− SD, * *p* < 0.05, ** *p* < 0.01.

**Figure 4:**
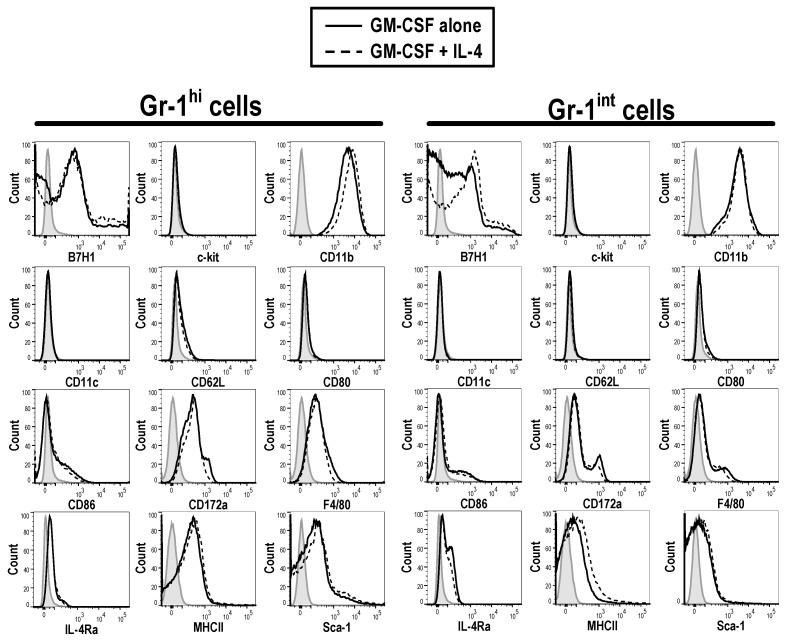
The phenotype of Gr-1^hi^ and GR-1^int^ cells from GM-CSF and GM-CSF + IL-4 derived BM cultures is consistent with the presence of MDSCs. Live cells from GM-CSF derived BM culture were characterized on day 3. Cells generated from BM with GM-CSF (10 ng/mL) (solid line) or GM-CSF (10 ng/mL) + IL-4 (5 ng/mL) (dotted line) were characterized with a panel of phenotypic markers, chemokine receptors and activation markers. The shaded grey area represents unstained control cells. Results shown are representative of three mice.

Within these cultures there were also a population of GM-CSF derived MDSCs identified. Upon analysis of GM-CSF derived Gr-1^hi^ and Gr-1^int^ MDSCs, they were found to have distinct morphologies (Supplementary Figure S1 and Figure S2) and growth patterns over time (Figure 3). Gr-1^hi^ and Gr-1^int^ MDSCs express high levels of CD11b (MFI 3827 ± 402.94), but not CD11c (MFI 31.27 ± 0.9), as well as low levels of other lineage markers such as CD62L (neutrophilic marker) (MFI 1157.33 ± 60.48), CD172α (MFI 523 ± 15.13) and F4/80 (myeloid markers) (MFI 533.33 ± 39.2) (Figure 4). Some MDSCs also expressed low levels of stem cell markers, c-kit (MFI 1126.67 ± 159.09) and Sca-1 (MFI 559.33 ± 27.79), suggesting that they are not just early-phase hematopoietic stem cells HSCs. The expression of these markers has been observed previously on MDSCs detected in tumor-bearing mice and cancer patients, suggesting that these cells are indeed the in vitro equivalents of the MDSCs found in the tumor microenvironment [6,24]. Furthermore, these MDSCs exhibit low levels of the activation markers CD80 (MFI 976.33 ± 93), CD86 (MFI 876.67 ± 37.63) and MHCII (MFI 478.67 ± 4.51). Apart from the different levels of Gr-1 expression (Gr-1^hi^, MFI 7071.33 ± 215.93; Gr-1^int^, MFI 820 ± 33.81), it was not possible to distinguish the two populations of MDSCs based on any other markers. There was also no detectable difference between the MDSCs generated from GM-CSF and GM-CSF + IL-4 cultures.

### 3.3. Functional Activation and Cytokine Secretion in Early GM-CSF Derived BM Cultures upon LPS Stimulation

**Figure 5:**
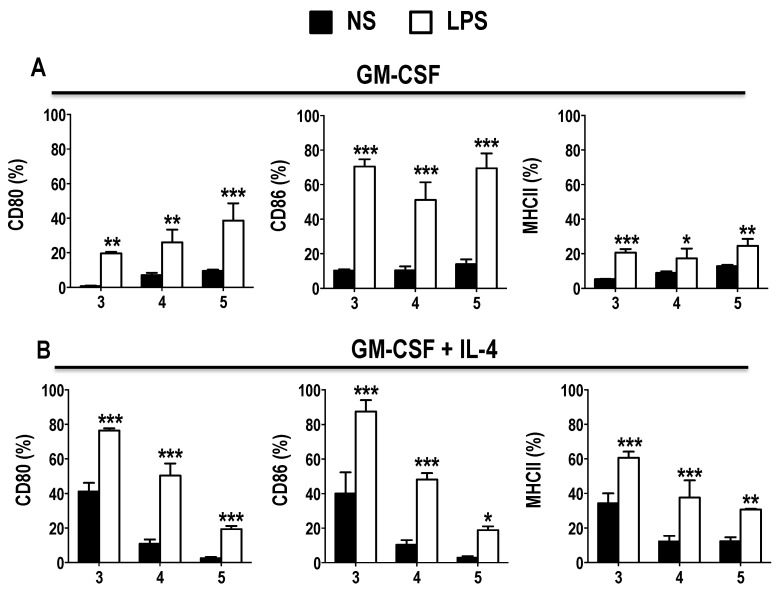
Expression of activation markers after stimulation with the TLR4 ligand LPS on DCs from early GM-CSF +/- IL-4 cultures. Day 3 to 5 DCs were stimulated with LPS for 24 h. The expression of CD80, CD86 and MHCII on (**A**) GM-CSF and (**B**) GM-CSF + IL-4 derived DCs were measured using flow cytometry. Results shown are from 3 mice. Two-way ANOVA was used to compare both experimental conditions at each time-point. Results displayed as mean +/− SD. * *p* < 0.05, ** *p* < 0.01, *** *p* < 0.001.

Next, it was examined whether the DCs generated in early GM-CSF cultures would be fully functional upon TLR stimulation at this early stage of development. To assess this, cells derived from GM-CSF BM cultures were stimulated on day 3 with the TLR4 agonist LPS for 24 h and expression of surface activation markers on the different cell subsets was analyzed by flow cytometry. Day 3 DCs generally expressed lower levels of the co-stimulatory marker CD80 compared to cells stimulated on days 4 or 5 of culture. However, day 3 DCs were able to up-regulate expression of activation markers such as CD80 from 0.68% ± 0.42 to 19.67% ± 0.84, CD86 from 10.25% ± 0.73 to 70.53% ± 4.14, and MHCII from 5.27% ± 0.19 to 20.67% ± 2.07 (Figure 5A). This up-regulation was similar to the percentage on day 4 (CD80, from 7.11% ± 1.33 to 25.97% ± 7.47; CD86, from 10.3% ± 2.35 to 51.2% ± 10.19; MHCII, from 8.91% ± 0.94 to 17.33% ± 5.65) and 5 (CD80, from 9.52% ± 0.72 to 38.7% ± 9.88; CD86, from 14% ± 2.77 to 69.5% ± 8.61; MHCII, from 12.87% ± 0.68 to 24.57% ± 4.05) DCs upon LPS stimulation (Figure 5A). This suggests that these early DCs are readily activated upon stimulation despite their relatively immature phenotype. Sheng et al., have also demonstrated that by day 6, DCs stimulated with zymosan (TLR2) or LPS (TLR4) could not reach the same level of activation marker expression (MFI) induced on DCs stimulated after three days in GM-CSF cultures [25]. GM-CSF derived DCs were further compared with DCs from GM-CSF + IL-4 cultures. It was noted that GM-CSF + IL-4 DCs upregulated CD80, CD86 and MHCII upon LPS stimulation. Furthermore, GM-CSF + IL-4 DCs expressed 41.17% ± 5.01 CD80, 40.67% ± 12.21 CD86 and 34.3% ± 5.72 MHCII (Figure 5B), which was higher than the GM-CSF derived DCs. However, co-stimulatory marker expression decreased over time in GM-CSF + IL-4 cultures, regardless if they were stimulated or not (Figure 5B). Whilst LPS stimulation significantly up-regulated expression levels, it did not restore them to the same expression levels observed on day 3 (Figure 5). Furthermore, co-stimulatory and activation marker expression on MDSCs (both Gr-1^hi^ and Gr-1^int^) was minimally altered following stimulation with LPS on days 3–5 (Supplementary Figure S3).

**Figure 6:**
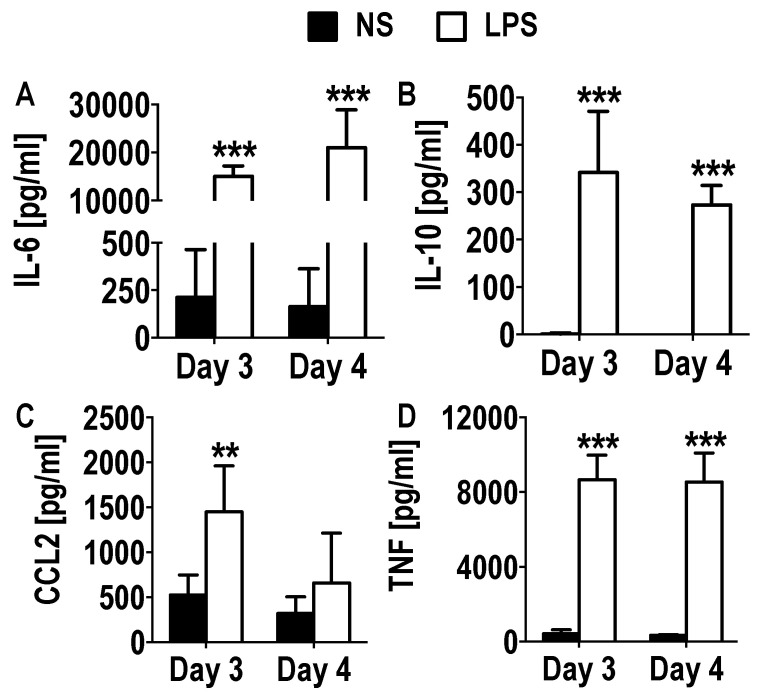
Cytokine section in early GM-CSF derived cultures upon LPS stimulation. Day 3 to 5 DCs generated from GM-CSF derived BM cultures were stimulated with LPS for 24 h. Culture supernatants were harvested after 24 h and CBA was used to measure the cytokine secretions within the culture, (**A**) IL-6, (**B**) IL-10, (**C**) CCL2, (**D**) TNF. Results shown are from three mice. Student’s t-Test was used to compare non-stimulated to LPS stimulated cultures. Results displayed as mean +/− SD. ** *p* < 0.01, *** *p* < 0.001.

Additionally, the cytokine secretion (IL-6, IL-10, CCL2 (MCP-1), TNF) from supernatants of day 3 and 4 GM-CSF derived cultures that were stimulated with LPS for 24 h were analyzed using a CBA. Large increases in the secretion of pro-inflammatory IL-6 (day 3, 15,042.44 pg/mL ± 2171.45; day 4, 21,103.06 pg/mL ± 7872.14) and TNF (day 3, 8661.14 pg/mL ± 1319.9; day 4, 8533.87 pg/mL ± 1560.76) were measured, as well as the anti-inflammatory cytokine IL-10 (day 3, 341.39 pg/mL ± 128.97; day 4, 273.37 pg/mL ± 40.86) (Figure 6). Furthermore, a smaller but significant increase in the chemokine CCL2 (day 3, 1450.8 pg/mL ± 509.98; day 4, 659.21 pg/mL ± 554.28) was measured (Figure 6). These results confirm that the cells in these early cultures are indeed functional. 

### 3.4. Early DCs Stimulate T Cells upon TLR4 Stimulation

**Figure 7:**
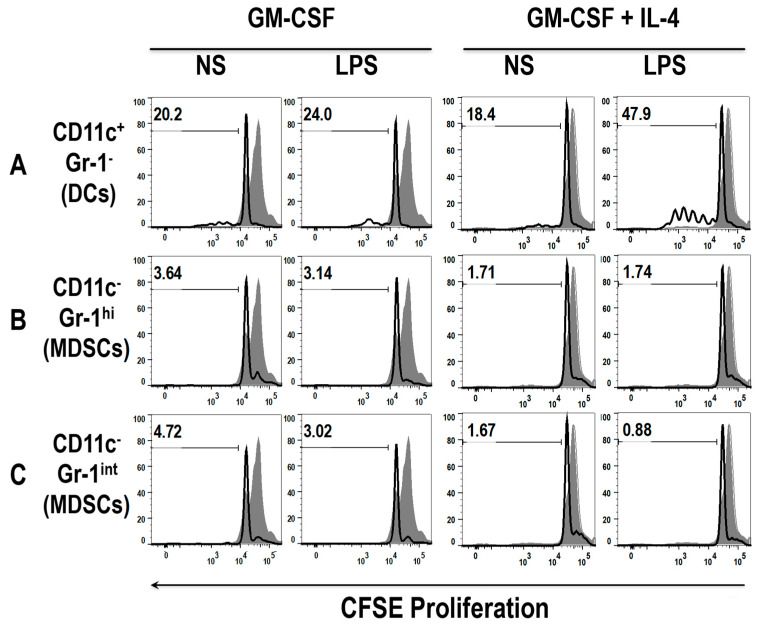
Early DCs, but not MDSCs, from both GM-CSF and GM-CSF + IL-4 cultures, induce T cell proliferation following LPS stimulation. Cells from day 4 GM-CSF derived BM cultures were sorted with a FACS Aria cell sorter into (**A**) DCs, (**B**) Gr-1^hi^ MDSCs and (**C**) Gr-1^int^ MDSCs. These cells were either un-stimulated or pre-stimulated with LPS for 24 h before incubation with T cells for 3 days at a ratio of 1:30 (DC: T cells). T cell proliferation was measured by CFSE. The shaded grey area represents the CFSE positive control. Numbers within the histograms depict percentage of cells proliferated. Histograms are representative of three mice.

To examine whether DCs and MDSCs harvested from the early cultures were able to induce T cells, a hallmark property of mature DCs, a mixed lymphocyte reaction (MLR) was used. Firstly, cells of day 3 or 4 GM-CSF or GM-CSF + IL-4 cultures were stimulated with LPS (1 μg/mL), or left unstimulated, for 24 h. They were then harvested and sorted for CD11c+ Gr-1- (DCs), CD11c- CD11b+ Gr-1^hi^ and CD11c- CD11b+ Gr-1^int^ (MDSCs) populations. Additionally, allogeneic T cells from the spleen of BALB/c mice were enriched using MACS^®^ columns. The sorted cells were then co-incubated with the enriched T cells (1 DCs: 30 T cells ratio) for a further 72 h and proliferation was assessed using CFSE staining (each cell division is indicated by a dilution of the CFSE dye). Early stage DCs from non-stimulated (NS) GM-CSF cultures induced T cell proliferation (Figure 7A, first panel), yet the Gr-1^hi^ and Gr-1^int^ MDSC populations did not (Figure 7B,C, first panel). Further investigation revealed that early GM-CSF derived DCs stimulated by LPS slightly increased their capacity to induce T cell proliferation (Figure 7A, second panel). Early MDSCs stimulated with LPS did not change, even decreased, their capacity to induce T cell proliferation (Figure 7B,C, second panel). Similar observations were detected with the GM-CSF + IL-4 derived DCs and MDSCs, where the GM-CSF + IL-4 DCs induced the greatest T cell proliferation, following LPS stimulation (Figure 7A, fourth panel). These results indicate that early GM-CSF derived DCs are capable of stimulating T cell proliferation, consistent with their heightened stimulatory phenotype following LPS stimulation. Conversely, the diminished ability of MDSCs to induce T cell proliferation is consistent with their lack of a stimulatory phenotype (Supplementary Figure S3). 

### 3.5. The Addition of LPS Enhances the Ability of Early MDSCs to Co-Stimualte T Cell Proliferation, in the Presence of Anti-CD3 and Anti-CD28

**Figure 8:**
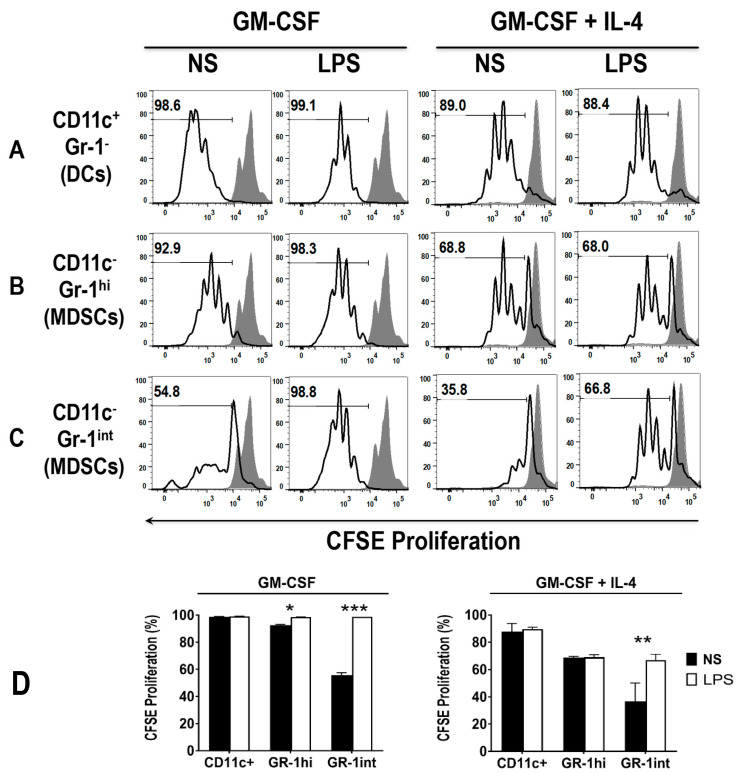
DCs and GR-1^hi^ MDSCs, but not GR-1^int^ MDSCs unless LPS stimulated, from both GM-CSF and GM-CSF + IL-4 cultures, co-stimulate T cell proliferation. Cells from day 4 GM-CSF derived C57BL/6 BM cultures were sorted with a FACS Aria cell sorter into (**A**) DCs, (**B**) Gr-1^hi^ MDSCs and (**C**) Gr-1^int^ MDSCs. These cells were either un-stimulated or pre-stimulated with LPS for 24 h before incubation with BALB/c T cells for 3 days at a ratio of 1:30 (DC: T cells) with the addition of anti-CD3 and anti-CD28 monoclonal antibodies (5 μg/mL each). T cell proliferation was measured by CFSE proliferation. The shaded grey area represents the CFSE positive control. Numbers within the histograms depict percentage of cells proliferated. Histograms are representative of two to three mice. (**D**) represents mean percentage of CFSE proliferation +/− SD of 2–3 mice. * *p* < 0.05, ** *p* < 0.01, *** *p* < 0.001.

As expected, DCs had a superior ability to induce T cell proliferation to MDSCs, which are known to have a suppressive role. To assess the ability of MDSCs to induce T cell proliferation in the presence of an added stimulus, and the effect of LPS on this function, cells of day 3 GM-CSF or GM-CSF + IL-4 cultures were stimulated, or not, with LPS (1 μg/mL) for 24 h. The cells were then harvested and sorted for Gr-1^hi^ and Gr-1^int^ MDSCs, as well as CD11c+ DCs using FACS. Additionally, allogeneic T cells from the spleen of BALB/c mice were enriched using MACS^®^ columns and co-incubated with the sorted DCs, as above, though in the presence of anti-CD3 and anti-CD28 (to stimulate T cells), for 72 h. The number of T cell divisions, in response to co-stimulation of anti-CD3/CD28 and either DCs or MDSCs was measured. Results showed that early MDSCs, particularly GM-CSF derived Gr-1^int^ MDSCs, do not co-stimulate T cells as readily as GM-CSF derived GR-1^hi^ MDSCs and DCs (Figure 8A–C, first panels, Figure 8D). 

Pre-treatment with LPS enhanced the co-stimulatory capacity of GM-CSF derived GR-1^int^ MDSCs, to the levels of GR-1^hi^ MDSCs and DCs (Figure 8A–C, second panels, Figure 8D). Interestingly, all GM-CSF + IL-4 derived cell subsets stimulated lower levels of T cell proliferation. The lowest levels of T cell co-stimulation in GM-CSF + IL-4 derived cells were observed with the GR-1^int^ MDSCs (Figure 8C, third panel, Figure 8D). Again, pre-treatment with LPS was able to enhance the co-stimulatory capacity of GR-1^int^ MDSCs, comparable to GR-1^hi^ MDSCs, but not to the level of DCs (Figure 8A–D). Whereas DCs and GR-1^hi^ MDSCs were able to strongly co-stimulate T cells in the presence of anti-CD3 and anti-CD28, GR-1^int^ cells displayed reduced co-stimulatory capacity and required an additional signal, i.e., LPS treatment, to acquire their co-stimulatory ability. These data suggest that the GM-CSF and GM-CSF + IL-4 derived MDSC subsets are fully functional in the early day 3-4 cultures.

### 3.6. Differential Uptake Capacity by Early GM-CSF Derived DCs and MDSCs

**Figure 9:**
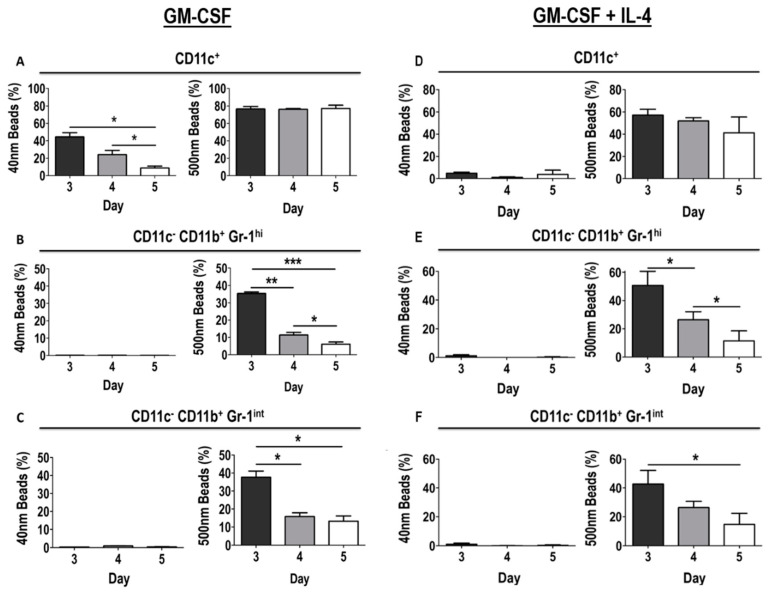
Differential uptake capacity of DCs and MDSCs in early GM-CSF and GM-CSF + IL-4 derived cultures. Day 3, 4 and 5 GM-CSF and GM-CSF + IL-4 derived BM cultures were incubated with 40 nm or 500 nm particles for one hour at 37 degrees °C in 5% humid CO_2_ atmosphere. Cells were harvested on respective days and endocytic capacity was analyzed by flow cytometry for both 40 nm and 500 nm particle uptake by (**A**,**D**) DCs, (**B**,**E**) Gr-1^hi^ MDSCs and (**C**,**F**) Gr-1^int^ MDSCs. Results are shown from three mice. One-way ANOVA was used to calculate statistical significance. Results displayed as mean +/− SD. * *p* < 0.05, ** *p* < 0.01, *** *p* < 0.001.

To assess another important function of DCs, endocytic capacity, cells from day 3, 4 and 5 GM-CSF derived BM cultures were incubated with fluorescent nanoparticles of two different sizes, 40 nm or 500 nm (to mimic small and larger pathogens, respectively), for one hour and uptake was assessed by flow cytometry. Both 40 nm and 500 nm fluorescent particles were taken up by GM-CSF derived DCs (Figure 9A). However, there was a significant decrease in the uptake of 40 nm fluorescent particles on day 4 and 5 (44.57% ± 4.18, 24.2% ± 4.88, 8.83% ± 2.22 on days 3, 4 and 5, respectively) (Figure 9A), suggesting that their endocytic capacity decreases as these DCs mature. Interestingly, uptake of 500 nm fluorescent particles by DCs remained constant throughout day 3 (76.57% ± 2.82), 4 (76.23% ± 0.75) and 5 (77.1% ± 3.74) (Figure 9A). 

In contrast, uptake of 40 nm particles was minimally detected in Gr-1^hi^ MDSCs (0.13% ± 0.007, 0.14% ± 0.005, 0.06% ± 0.02 on days 3, 4 and 5, respectively), and Gr-1^int^ MDSCs, where a very small percentage of cells tested positive for uptake of 40 nm particles (0.35% ± 0.01, 0.94% ± 0.15, 0.48% ± 0.04 on days 3, 4 and 5, respectively), though no significant changes were observed over time (Figure 9B). Both Gr-1^hi^ and Gr-1^int^ MDSCs were able to ingest 500 nm particles efficiently (Figure 9B). Unlike DCs, the capacity of MDSCs to take up 500 nm particles was significantly reduced in cells from day 4 (11.39 ± 1.52 and 15.83% ± 2.14 for Gr-1^hi^ and Gr-1^int^ MDSCs, respectively) and day 5 (6.09% ± 1.24 and 13.27% ± 2.9 for Gr-1^hi^ and Gr-1^int^ MDSCs, respectively) cultures when compared to cells from day 3 cultures (35.37% ± 0.91 and 37.67% ± 3.47 for Gr-1^hi^ and Gr-1^int^ MDSCs, respectively) (Figure 9B,C).

Similar results were seen when analyzing the uptake capacity of early DCs and MDSCs from GM-CSF + IL-4 derived BM cultures. In contrast to GM-CSF only cultures, where 44.57% ± 4.81 of early DCs had taken up 40 nm particles, only 4.84% ± 1.05 of early DCs derived from GM-CSF + IL-4 cultures tested positive for 40nm bead uptake on day 3 (Figure 9D). The uptake did not significantly change in DCs from day 4 and 5 cultures (Figure 9D). Furthermore, a small, but not significant, reduction in the uptake of 500 nm particles was observed for DCs on day 4 and 5 when compared to day 3 cultures (Figure 9D). Both Gr-1^hi^ and Gr-1^int^ MDSCs derived from GM-CSF + IL-4 cultures showed minimal uptake of 40 nm particles in a very small proportion of cells from day 3 (1.21% ± 0.83 and 1.06% ± 0.79, respectively), day 4 (0.04% ± 0.005 and 0.08% ± 0.03, respectively) and day 5 (0.22% ± 0.32 and 0.27% ± 0.37, respectively) cultures (Figure 9E). Similarly to their GM-CSF derived counterparts, Gr-1^hi^ and Gr-1^int^ MDSCs derived from GM-CSF + IL-4 cultures were able to take up 500 nm particles (50.67% ± 9.98 and 42.7% ± 9.44 on day 3, respectively, Figure 9E,F) and, furthermore, their capacity to take up these particles decreased over time (26.4% ± 5.68 and 26.4% ± 4.25 on day 4, 11.4% ± 7.15 and 14.66% ± 7.7 on day 5, respectively) (Figure 9E,F).

## 4. Discussion

DCs are renowned for their potent antigen-presentation properties and have been extensively studied over the last decade as targets in immunotherapies and vaccines [26,27,28]. Given many studies on DCs and most DC immunotherapies use GM-CSF derived culture systems, it is of interest to understand the characteristics of these types of cultures further. Most studies generate DCs in vitro from human peripheral blood or murine BM. They are often generated using GM-CSF alone or with a mixture of additional cytokines such as IL-4, and additional maturation agents such as IL-6, TNF, IFN-γ or TLR agonists for a period of time if using for immunotherapies [10,20,29]. Such extended cultures can increase the yield of DCs with a mature phenotype, which is ideal for inducing an immune response [20,29]. The current study has shown that the crucial period for DC generation occurs between day 3 and 4 in GMCSF derived cultures using cells that are derived from HSCs. These early GM-CSF DCs displayed a conventional DC phenotype (B220-CD4-CD8-CD11b+CD11c+) and expressed lower levels of co-stimulatory molecules when compared to later-stage DCs (> day 4) or GM-CSF + IL-4 derived DCs. In addition, they were capable of upregulating co-stimulatory molecules and activating T cells upon stimulation with LPS, as well as displaying functional endocytic capacity. These data show that early GM-CSF derived DCs display the important properties of their more mature counterparts and thus may present an interesting target for immunotherapies. Although GM-CSF is primarily used to generate in vitro DCs, these cultures do not only contain the desired DCs, but also other myeloid cell types [14,30]. 

In recent studies, GM-CSF has been shown to generate a suppressive myeloid cell subset, MDSCs [2,3,4]. MDSCs are a mixture of early myeloid progenitors, such as immature granulocytes, macrophages, and DCs [2,3]. Their phenotype and functions have been studied in detail only more recently [31,32]. Whilst some studies use CD11b+, GR-1+ phenotype to describe MDSCs in different disease models [33,34,35], others define granulocytic MDSCs as Ly6Ghi and monocytic MDSCs as Ly6Chi [36,37]. Here it is shown that GR-1^hi^ MDSCs are morphologically granulocytic, confirmed by their expression of Ly6G, and GR-1^int^ MDSCs are monocytic like cells morphologically and express high levels of Ly6C. This is consistent with other reports of GR-1 and Ly6C/G expression [37,38]. MDSCs are potent suppressor cells, and their presence in various pathological conditions has been proven to cause damaging effects and worsen prognosis in patients [39]. The present data show that MDSCs are generated in vitro alongside early DCs in the GM-CSF and GM-CSF + IL-4 cultures. Early MDSCs do not have the same ability to stimulate T cells, compared to DCs. Though it has also been shown that these early MDSCs are functional in terms of their ability to co-stimulate T cell proliferation in the presence of anti-CD3 and anti-CD28, but not without this extra signal. Interestingly, this effect of GR-1^int^ MDSCs could be enhanced upon LPS stimulation. Whether this ability is correlated with their suppressive capacity is yet unknown. 

Previous studies have shown that early MDSCs lose their anti-inflammatory state in septic mice [40]. Additionally, TLR stimulation has been shown to block the suppressive effects of MDSCs in tumor-bearing mice [41], indicating that MyD88 signaling may play a role in the abrogation of the suppressive effects of MDSCs. Similarly, Greifenberg et al., analyzed in vitro GM-CSF cultures stimulated with LPS and IFN-γ and showed a higher level of nitric oxide production [42]. Stimulation with LPS diminished the T cell suppressive capacity in the culture [42]. Interestingly, in their study they assessed in vivo MDSC populations and found two splenic populations (Gr-1+, CD11b+ cells) that have MDSC phenotypes and a capacity to suppress T cells in vitro [42]. Bunt et al., analyzed blood MDSCs from mice with mammary tumors, which induces an inflammatory environment, and found that in the presence of IFN-γ and LPS, the mice produced more IL-10 and increased expression of CD14 on MDSCs, which was TLR4 dependent [43]. Others have assessed the influence of chemotherapy (Paclitaxel) on GM-CSF cultures, and observed that low level paclitaxel did not interfere with their generation, instead it increased DC numbers in the culture, though in a TLR4 independent manner [44]. Hong et al. described the importance of MyD88 in MDSC-induced NADPH oxidase and arginase-1 production [45]. MyD88 knockout MDSCs failed to induce suppression in tumor-bearing mice, which resulted in an immunogenic response, followed by a significant reduction in tumor mass [45]. This suggests that with the use of appropriate conditions to grow the DCs used for a vaccine or immunotherapeutic treatment, it might be possible to reduce or eliminate the suppressive effects of MDSCs and ensure that the early DCs are sufficiently activated to stimulate a potent T cell response. 

In future it would be important to not only assess MDSCs T cell suppressive ability, but also measure the production of cytokines (i.e., IL-10) and analyze the levels of arginase 1 and ROS and NO production in the cultures. Whilst the present study has not examined the cytokine profile of cells derived from GM-CSF + IL-4 cultures, similar studies have shown TNF-α and IL-12p70 secretion in rat BM cultures of both GM-CSF and GM-CSF + IL-4 cultures [46]. Additionally, upon stimulation with LPS, both cytokines were increased more in GM-CSF + IL-4, compared to GM-CSF alone, cultures [46]. Future studies comparing these cultures should expand the panel of cytokines analyzed, both with and without LPS stimulation. Other cells in the culture may also contribute to the cytokine profile, therefore future studies may also look for the presence of F4/80+ macrophages. As these cells are strongly adherent (not collected for the above study) [15], they were not expected to be present in high numbers in this study. 

Furthermore, this study investigated the endocytic capacity of early DCs and MDSCs generated from GM-CSF derived BM cultures. The initial endocytic capacity of the GM-CSF derived early DCs and MDSCs was assessed by incubating with 40 nm and 500 nm particles for one hour and noted that the early DCs could take up 40 nm and 500 nm particles. However, the uptake ability of the 40 nm particles decreased over two days of maturation, whereas uptake of 500 nm particles was maintained. Previous studies have shown that BM derived DCs take up 40 nm particles utilizing the caveolar endocytic pathway, whereas 500 nm nanoparticles are taken up by phagocytosis [47]. Extending the incubation time to 24 h for the particles with the cells showed a small but significant difference in the 40 nm and 500 nm particle uptake in DCs over time (data not shown). This suggests that while these early DCs are capable of taking up particles of both sizes, it is their rate of uptake of 40 nm particles that is affected as they mature. This was further confirmed by the fact that early GM-CSF + IL-4 derived DCs (the mature equivalent of GM-CSF derived DCs) did not effectively take up 40 nm particles after one hour of incubation. Other studies have shown that immature DCs have higher endocytic capacity and that maturation has a specific downregulation effect on phagocytosis and macropinocytosis [48,49]. Interestingly, these studies also showed that DC maturation did not affect uptake of nanoparticles by certain mechanisms, such as receptor mediated endocytosis [48,49]. The heterogeneity in DC subsets and type of activating signal may also influence the phagocytic ability of DCs, with CD40 ligand-activated cells retaining phagocytic and presentation capabilities, though these processes were diminished in LPS-activated cells [50].

When the uptake of MDSCs was investigated, the data showed that both MDSC subsets preferred 500 nm particles, although some cells took up 40 nm particles. This was consistent with early MDSCs derived from both GM-CSF and GM-CSF + IL-4 cultures. Both cultures showed a similar trend of a decrease in uptake of the 500 nm particles over time, which suggests that MDSCs generated from both GM-CSF cultures and GM-CSF + IL-4 cultures are similar in terms of maturity and function. Given the distinct preferences in particle size uptake by early DCs and MDSCs, it suggests different kinetics of endocytosis in the different cell types. Future studies would benefit from analyzing the influence of LPS stimulation on endocytosis in both DC and MDSC populations.

## 5. Conclusions

Taken together, the data presented here reveal information on the type of cells and their function in early GM-CSF derived BM cultures. This may have additional potential value for early GM-CSF-derived DCs for their use in vaccine models and immunotherapies. Further investigation is required to fully comprehend and effectively manipulate these cultured cells. The data discussed show that cells harvested from early GM-CSF (as well as GM-CSF + IL-4) derived BM cultures yield functional DCs and MDSCs, which are useful tools to study maturation, activation and T cell stimulation capabilities, as well as endocytic capacity, within these cultures. Given their higher endocytic capacity, early DCs may also be a promising candidate for future immunotherapies, though much more research would be needed to confirm this direction. 

## Figures and Tables

**Table 1:** List of flow cytometry antibodies.

Antibody	Clone	Fluorophore	Company	Catalogue Number
B220	RA3-6B2	FITC	BD Biosciences	553088
B7H1	MIH5	PE	BD Biosciences	557495
c-kit	2B8	FITC	BD Biosciences	553354
CCR4	2G12	PECy7	BioLegend	131214
CCR5	HM-CCR5	APC	BioLegend	107011
CCR7	4B12	PerCP Cy5.5	eBioscience	45-1971-82
CD103	M290	PE	BD Biosciences	557495
CD11b	M1/70	PE	BD Biosciences	553311
CD11b	M1/70	PECy7	BD Biosciences	552850
CD11c	HL3	V450	BD Biosciences	560521
CD14	rmC5-3	FITC	BD Biosciences	553739
CD172a	P84	APC	BD Biosciences	560106
CD205	205yekta	PECy7	BD Biosciences	25-2051-41
CD205	NLDC-145	Biotin	Cederlane	CL89145B
CD24	M1/69	PECy7	BD Biosciences	560536
CD3	17A2	FITC	BD Biosciences	555274
CD3	500A2	AlexaFluor 700	BD Biosciences	557984
CD31	MEC13.3	APC	BD Biosciences	551262
CD4	GK1.5	PE	BD Biosciences	557308
CD4	RM4-5	PerCP	BD Biosciences	553052
CD40	3/23	FITC	BD Biosciences	553791
CD44	IM7	Biotin	BD Biosciences	553132
CD62L	MEL-14	FITC	BD Biosciences	553150
CD80	16-10A1	Biotin	BD Biosciences	553767
CD86	GL1	PE	BD Biosciences	553692
CD8a	53-6.7	PE	BD Biosciences	553033
CD8a	53-6.7	PerCP	BD Biosciences	553036
CD8a	53-6.7	APC	BD Biosciences	553035
F4/80	BM8	PE	eBioscience	12-4801-80
Gr-1	RB6-8C5	PerCP Cy5.5	BD Biosciences	552093
Gr-1	RB6-8C5	Biotin	BD Biosciences	553125
IL-4Ra	mIL4R-M1	PE	BD Biosciences	561695
MHCI class II	M5/114.15.2	APCCy7	BD Biosciences	47-5321-80
NK1.1	PK136	FITC	eBioscience	12-5941-83
Sca-1	D7	PE-Cy7	BD Biosciences	558162
Streptavidin		AlexaFluor 700	Invitrogen	S21383
Streptavidin		APC	BD Biosciences	554067
Streptavidin		APCCy7	eBioscience	47-4317-82
Streptavidin		FITC	BD Biosciences	554060
Streptavidin		PE	BD Biosciences	554061

**Table 2:** Comparison between the expression levels of phenotypic markers, chemokine receptors and activation markers on CD11c+ cells.

Surface Markers	GM-CSF DCs	GM-CSF + IL-4 DCs	*p*-Value	Significance
	Mean MFI ± SD	Mean MFI ± SD		
CD11b	14,969 ± 2668	4072 ± 473.1	0.0233	*
CD44	75,858 ± 5882	52,938 ± 3483	0.0038	**
CD80	751.3 ± 10.79	1368 ± 13.5	<0.0001	***
CD86	991 ± 26.96	3229 ± 427	0.0114	*
CD103	4933 ± 253.2	2444 ± 153.3	0.0029	**
CD172α	1943 ± 65.59	1599 ± 113.4	0.0098	**
F4/80	1499 ± 124.2	733.3 ± 48.68	0.0049	**
MHCII	2052 ± 248.6	12,634 ± 452.3	0.0004	***
Sca-1	3499 ± 339.2	2892 ± 278.9	0.007	**

Results shown are from three mice. Unpaired students T test was used to compare groups. Results displayed as mean +/− SD. * *p* < 0.05, ** *p* < 0.01, *** *p* < 0.001. DCs, dendritic cells; MFI, Mean fluorescence intensity; SD, standard deviation.

## Supplementary Materials

The following are available online at https://www.mdpi.com/2076-393X/8/3/522/s1, Figure S1: Morphology of early stage DCs and MDSCs. Morphology of sorted cell populations; CD11c+ resembling DCs, Gr-1^hi^cells resembling granulocytes (termed, granulocytic MDSCs); Gr-1^int^ cells resembling monocytes (termed monocytic MDSCs), Figure S2: Expression of Ly6C and Ly6G on CD11b+, GR-1+ MDSCs. BM cells were cultured with GM-CSF for 3 days and phenotype of CD11c-, CD11b+, GR-1+ MDSCs assessed by flow cytometry. (A) Percentage of CD11c-, CD11b+ cells that are GR-1^hi^ and GR-1^int^. (B) MFI of Ly6G and Ly6C on GR-1^hi^ and GR-1^int^ cells. GR-1^hi^ cells express both Ly6G and Ly6C, whereas GR-1^int^ cells only express Ly6C. (C). Representative histograms of Ly6C and Ly6G with GR-1^hi^ and Gr-1^int^ overlays. *n* = 2 mice, Figure S3: Expression of co-stimulatory markers on early (D3–5) GM-CSF derived MDSCs upon LPS stimulation. Day 3–5 GM-CSF derived BM MDSCs were stimulated with LPS for 24 h prior to harvesting. The expression of the maturation and activation markers CD40, CD80, CD86 and MHCII were analyzed using flow cytometry. The shaded grey area represents unstained control cells, solid black line represents unstimulated cells and dotted black line represents cells stimulated with LPS. Results shown are representative dot plots of three mice.
