# Supplementary material for: Dendritic Cells and Myeloid Derived Suppressor Cells Fully Responsive to Stimulation via Toll-Like Receptor 4 Are Rapidly Induced from Bone-Marrow Cells by Granulocyte-Macrophage Colony-Stimulating Factor
Source: Vaccines (Basel). 2020 Sep 12;8(3):522. doi: 10.3390/vaccines8030522 (PMC7564202; doi:10.3390/vaccines8030522)
Supplement: Supplementary file 1 [file vaccines-08-00522-s001.pdf]

vaccines

Article

Dendritic Cells and Myeloid Derived Suppressor Cells Fully Responsive to Stimulation via Toll-Like Receptor 4 Are Rapidly Induced from Bone-Marrow Cells by Granulocyte-Macrophage Colony-Stimulating Factor

Ying Ying Kong [1], Kirsty Wilson [2], Vasso Apostolopoulos [3,*,†] and Magdalena Plebanski [2,*,†]

Supplementary Materials

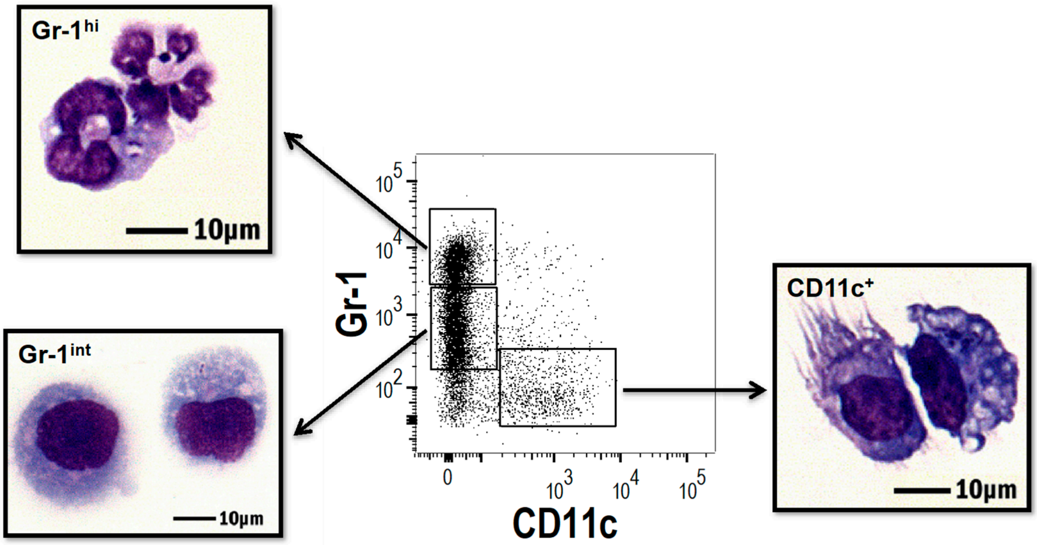

Figure S1. Morphology of early stage DCs and MDSCs. Morphology of sorted cell populations; CD11c+ resembling DCs, Gr-1^{hi} cells resembling granulocytes (termed, granulocytic MDSCs); Gr-1^{int} cells resembling monocytes (termed monocytic MDSCs).

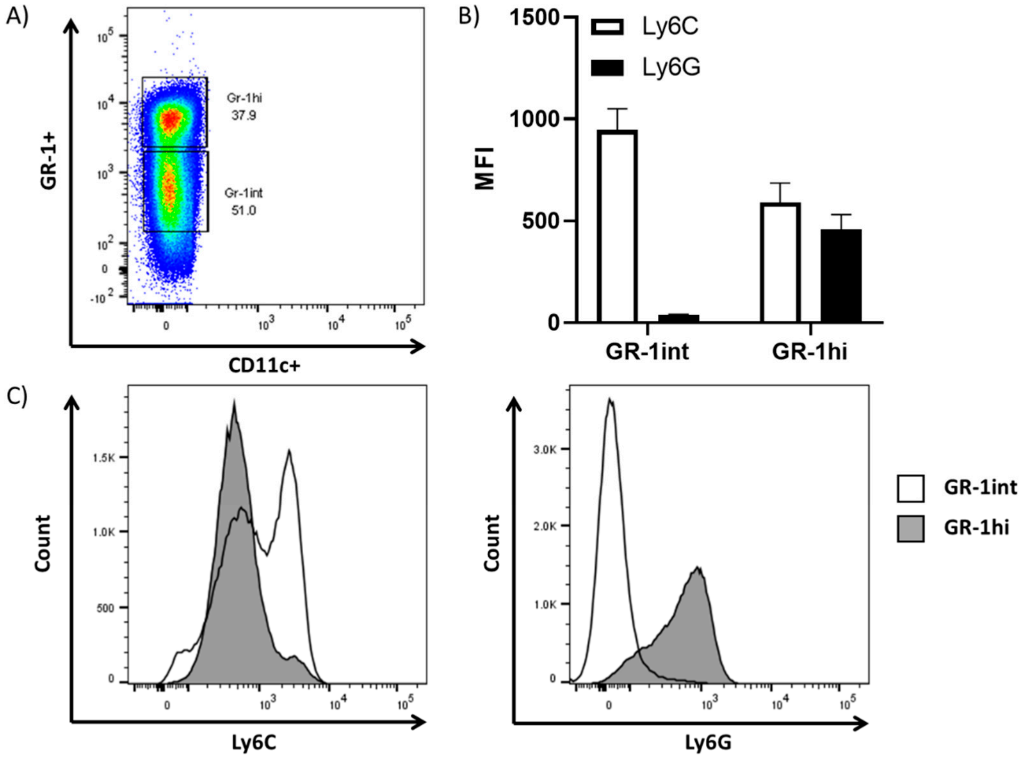

Figure S2. Expression of Ly6C and Ly6G on CD11b+, GR-1+ MDSCs. Bone marrow cells were cultured with GM-CSF for 3 days and phenotype of CD11c-, CD11b+, GR-1+ MDSCs assessed by flow cytometry. (A) Percentage of CD11c-, CD11b+ cells that are GR-1^{hi} and GR-1^{int}. (B) MFI of Ly6G and Ly6C on GR-1^{hi} and GR-1^{int} cells. GR-1^{hi} cells express both Ly6G and Ly6C, whereas GR-1^{int} cells only express Ly6C. (C). Representative histograms of Ly6C and Ly6G with GR-1^{hi} and Gr-1^{int} overlays. n = 2 mice.

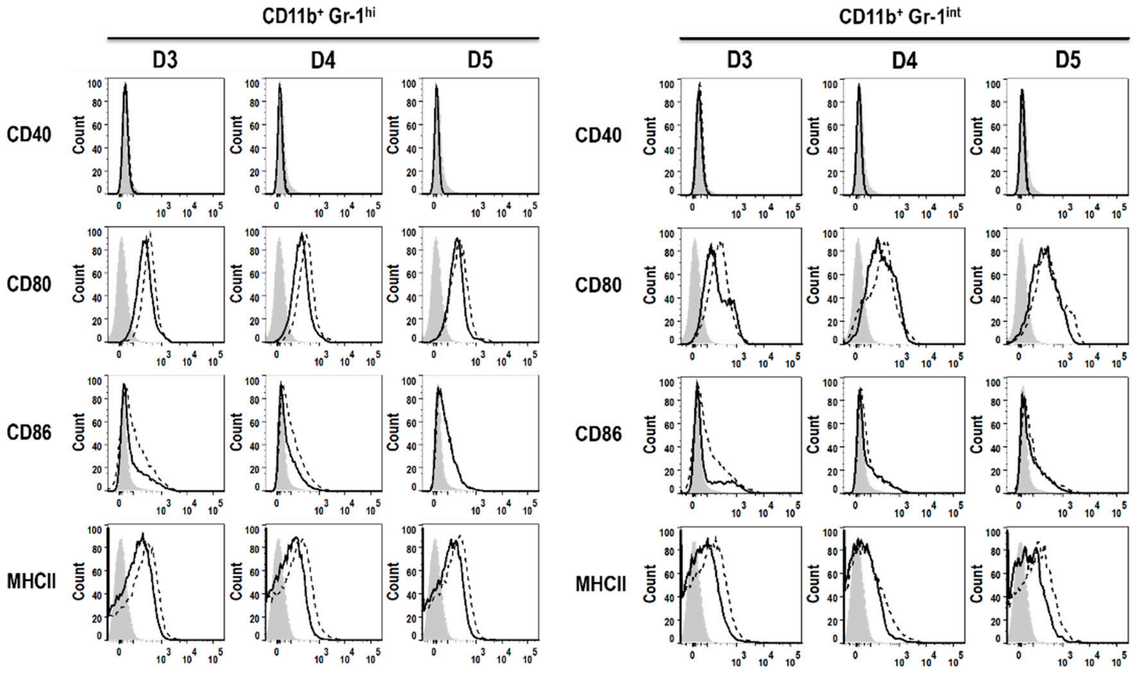

Figure S3. Expression of co-stimulatory markers on early (D3–5) GM-CSF derived MDSCs upon LPS stimulation. Day 3–5 GM-CSF derived BM MDSCs were stimulated with LPS for 24 h prior to

harvesting. The expression of the maturation and activation markers CD40, CD80, CD86 and MHCII were analyzed using flow cytometry. The shaded grey area represents unstained control cells, solid black line represents unstimulated cells and dotted black line represents cells stimulated with LPS. Results shown are representative dot plots of three mice.

© 2020 by the authors. Submitted for possible open access publication under the terms and conditions of the Creative Commons Attribution (CC BY) license (http://creativecommons.org/licenses/by/4.0/).
